# Responsible innovation: Its role in an era of technological and regulatory transformation

**DOI:** 10.1049/enb2.12005

**Published:** 2021-03-29

**Authors:** Joyce Tait, Alex Brown, Isabela Cabrera Lalinde, Daniel Barlow, Matthew Chiles, Paul Mason

**Affiliations:** ^1^ Innogen Institute University of Edinburgh High School Yards Edinburgh UK; ^2^ Strathclyde Institute of Pharmacy and Biomedical Sciences University of Strathclyde Glasgow UK; ^3^ BSI London UK; ^4^ Innovate UK Swindon UK

**Keywords:** biotechnology, BSI standards, decision making, government policies, innovation management, organisational aspects, supply chains

## Abstract

In the development of innovative technology products, companies of all sizes are being encouraged to innovate responsibly and regulators are encouraged to adapt their regulatory systems to be smarter, more proportionate and adaptive to the needs of innovative technologies. The British Standards Institution Responsible Innovation (RI) Guide (Publicly Available Specification [PAS] 440) is an industry‐wide standard relevant to both these policy trends. It supports companies by providing a framework to demonstrate the balance between the potential benefits and harms and, if necessary, to take action to maximise the benefits and/or minimise the harms. It includes guidance on engagement with stakeholders and will codify what stakeholders can expect from companies undertaking responsible innovation, paving the way to more harmonious relationships among stakeholders with differing interests and values. A cross‐sectoral survey of innovative companies showed that 90% favoured the development of such a standard. PAS 440 was also trialled in two early‐stage biotechnology companies and its expected benefits included contributing to coordinated responsible behaviour along a supply chain; better company and stakeholder understanding of the product properties; supporting decision‐making on whether or not to start a company; considering the risks of not developing the product and avoiding reputational risks. Benefits were expected to be increasingly significant as the RI standard becomes widely adopted.

## 1 INTRODUCTION

The innovation ecosystem for products of engineering biology is complex and rapidly evolving and companies face a daunting array of challenges in guiding their products to market. As in all other sectors of the economy, they will need to find a viable, resilient and adaptive business model and a place for their product in an existing supply chain or in some cases devise a new business model and even a new supply chain serving a new market.

Additionally, regulatory systems can be more time‐consuming and expensive than for many other sectors, creating significant barriers to entry for innovative small companies, particularly those lacking capital or resource and developing transformational products that would be disruptive of the business models of the current companies [1]. Many of today's regulatory systems are based on those introduced in the 20th century for technologies that are very different from innovative biotechnologies, and innovators are nevertheless expected to adapt their innovations to meet the needs of these regulatory systems [2]. However, a new ethos is emerging whereby regulators are increasingly willing to adapt their regulatory systems to help the society benefit safely from innovative technologies [3] as has been the case with the recent regulatory fast tracking of vaccines against COVID‐19, based on synthetic biology. Future regulatory systems across the board are expected to be more proportionate and adaptive to the risks and benefits of new technologies. They will be smarter and more targeted, rather than involving a lowering of standards for safety, quality and efficacy, and standards are expected to play a major role in the future regulatory reform [2]. Regulators are increasingly interested in exploring better ways to ensure public safety is maintained while not delaying beneficial innovation and have received government support for such initiatives [4].

In accordance with this regulatory reform agenda, since pre‐2000s, there have been calls for more ‘responsible’ research and innovation (RRI) [5], supported by generous research funding schemes, particularly the EU's Horizon 2020 programme. Most of the projects funded by these initiatives have focused on responsible research (RR) rather than responsible innovation (RI) and until very recently, there has been a lack of guidance for companies on how they are expected to innovate responsibly [6].

These parallel initiatives, RR and RI, are not independent. An important aspect of RI is the need for companies to engage with public stakeholders and citizens to ensure that their needs and desires are being taken into account in the innovation process, and ensuring that innovative technologies will be effectively regulated is a recurring theme in most engagement initiatives. Successful regulatory reform will therefore need to go hand in hand with a greater focus from companies on demonstrating that they are innovating responsibly.

This article makes the case for a standard to support companies in delivering RI and in demonstrating that they have done so, and describes the development and application of the British Standards Institution (BSI) Responsible Innovation Guide, PAS 440 [7], also referred here as the RI standard. A Publicly Available Specification (PAS) is a type of BSI standard that is commissioned by an external sponsor and developed rapidly to respond to an immediate business need. A ‘Guide’ gives less prescriptive advice than some other forms of standard and reflects the current thinking and practice amongst experts in a subject.

We describe the experience of trialling the RI standard on two cases involving small companies at the start of their innovation journey, and draw some conclusions relevant to its future development.

## 2 BUILDING A CASE FOR A RESPONSIBLE INNOVATION STANDARD

Compared to responsible research, different issues will arise at later developmental stages for a product, and different actors and stakeholders will be involved. Also, decisions will need to be made on timescales that reflect the real challenges faced by companies in a competitive economic environment. The Technology Strategy Board (TSB, now Innovate UK), in 2012, was the first UK funding body to apply RI principles to companies applying for public funding for translational research projects in synthetic biology [8]. The TSB Responsible Innovation Framework (RIF) aimed ‘… to fund projects where the ‘anticipated commercial use’ of the project outcomes meets, on the balance of positive and negative drivers, the standards outlined … for responsible innovation’; and ‘… to help companies ***anticipate and give responsible consideration*** to the ***intended*** and ***potential unintended*** impacts of the commercial development and use of the technology, including the potential for misuse, ***before the work begins*** *’* (TSB emphases).

The TSB's RIF reflected the organisation's role as a public sector funder, and was based on (i) positive drivers (factors in favour of supporting projects), (ii) negative drivers (factors against supporting projects) and (iii) regulatory drivers. In PAS 440, the perspective shifts to that of the innovating company.

However, further development of this approach to the RI standard development hit a roadblock at about this time. It was initially applied only to those working on synthetic biology‐related innovation and companies were understandably concerned that theirs should not be the only sector required to demonstrate that it was innovating responsibly. Other innovation programmes run by the TSB/Innovate UK at that time invested in areas where the societal implications were better understood, and there was felt to be no need to deploy the RIF in such areas. Agreement among companies on the need for a standard is an essential prerequisite for its development.

In the intervening years, pressure for companies to demonstrate responsible innovation has increased and become more pervasive influenced, among other things, by evidence of questionable data use by some data analytics companies [9] and by questions being raised about breakthrough technologies like facial recognition, other uses of AI, robotics and driverless vehicles.

The 2017 PAGIT report [2] recommended the development of an RI standard in the context of regulatory reform, and this coincided with a more general company understanding of the value of being able to demonstrate that they are innovating responsibly, for example [7, p. iv]:

- long‐term cost and risk reductions;
- more resilient new product/service offerings to potential customers;
- improvement of societal trust in the company and maintenance of social licence to operate;
- improved relations with investors and greater investor confidence in the company;
- greater attractiveness as an employer;
- better supply chain relationships;
- improved reputation and brand value;
- increased innovation capabilities;
- improved ability to communicate the value of products and services to investors, companies, customers and citizens;
- better relationships with governments, regulators and local communities; and
- improved capacity for long‐term planning and sustainability.

## 3 DEVELOPING PAS 440

### 3.1 Scoping survey to assess demand

The first stage in the development of PAS 440 was a survey, conducted through BSI, of companies that were potential users of such a standard. This was a critical point for the project. If there was no enthusiasm for it, it would have been harder to justify going ahead. The aim of the survey was to assess the appetite in industry to use an RI standard; provide an indication of what ‘responsible innovation’ would mean to the companies; gain insight on what it should cover; and explore any differences between the innovation areas.

Considerable thought was given to selecting whom to interview, to learn the perspectives of businesses of different sizes, operating in a range of technology and market areas. The in‐depth interview‐based survey involved 48 companies in the following sectors: AI and big data, 13; life sciences, 7; robotics and autonomous systems, 7; social media, 7; advanced materials, 7; and others, 7. There was good representation of both companies operating a business‐to‐business model and of those with a business‐to‐consumer model. Forty‐three companies (90%) considered the development of the RI standard to be quite important or very important.

At the same time as this survey, Doteveryone undertook a similar questionnaire‐based survey of 1010 technology professionals that included a question from BSI/Innovate UK: ‘How interested are you in the idea of a single framework for the responsible governance of innovation, that lays out the basic principles to be considered, and that could be used by companies of all size for any technology in any market?’ On answering this question, 78% of respondents were either very interested or fairly interested.

Quotes from the companies involved in this survey provided guidance on how they would like to see the RI standard developed, covering the following areas:

- the current lack of a framework to help companies to ‘… do the right things for the right reasons’;
- encouraging companies to think about what could go wrong;
- countering current trends towards public misinformation;
- acting as a checklist of key issues with a practical, rather than a theoretical focus;
- encouraging companies to think about the public good aspects of their innovations;
- it should be as dynamic, flexible and adaptable as the technologies it embraces;
- companies should be able to demonstrate compliance, that is, it should be an accreditable standard; and
- it should be broad enough to cover all potential use cases but specific enough to be useful.

There was also a positive response to the involvement of Innovate UK in the development of the RI standard, including that it ‘is the most natural standard‐bearer for this framework’, is ‘setting the innovation agenda in the UK’ and ‘sends the right signals to those working with innovative technologies’.

Most of the minority negative opinions expressed by companies in the survey related to the potential risk that a poorly designed PAS might become overly burdensome and stifle innovation. As these companies emphasised, it should be an enabler, not a barrier to innovation, it should be easy to implement and not the ‘… place where some innovation goes to die’, and it should not exacerbate the UK's current risk aversion where we will ‘… occupy the moral high ground but go bust in the process’.

The challenge for those developing the PAS was thus to deliver a framework that supports companies in behaving responsibly, enables them to communicate widely that they are behaving responsibly and at the same time adds value to their business, and is manageable within the resource constraints of operating in fiercely competitive environments.

### 3.2 Delivering on expectations

Insights gained from earlier research, particularly the PAGIT report funded by BEIS/BSI [10], had already identified many of the issues raised during the PAS 440 survey and had led to proposals for developing an RI approach that is proportionate to the needs of companies developing innovative technologies. The original TSB RIF took a similar approach in 2012, so the approach is well recognised and widely supported.

The following features, building on the TSB work, refined during the PAGIT project and consolidated in PAS 440, are intended to ensure that the RI process is manageable, even for small companies, by being proportionate to the needs and properties of the innovative technologies involved, their stage of development and the likely the extent of stakeholder interest. The RI standard has the potential to codify what members of the public can reasonably expect from companies when they advocate responsible innovation and hence pave the way to more harmonious relationships between stakeholders with differing interests and values. As explained in the preface to PAS 440 [7, p ii], 440 Hz is ‘concert pitch’, the frequency of the note to which the whole orchestra tunes to ensure that they play in harmony. The RI standard will perform a similar function so that innovation delivering new products, services and processes will work for all parts of a supply chain, and be accepted by a wider society.

PAS 440, as a practical guide, is particularly targeted to transformative (disruptive) innovations across a broad range of business sectors, including life sciences. It attempts to be realistic about the demands on company resources (time and money) in often fiercely competitive environments. It also aims to support companies in understanding what is needed to deliver RI at different stages in the development process, for different types of innovation across all developmental stages up to and beyond the market launch and gives guidance on charting a path through the potentially conflicting aspirations and demands of a broad range of different stakeholders.

Several important features set this RI standard apart from other approaches to RI.

1. It sets out basic principles to support company decision‐making, particularly where there are multiple stakeholder interests and values. A clear distinction is made between principles that are relevant to the behaviour of the company as a whole (e.g. respect for international norms of behaviour and for human rights), and principles that are relevant to the development of specific innovations. Innovation‐specific principles include the EU Precautionary Principle which is relevant to situations where there is uncertainty about future benefits and risks of an innovation [11] and the EU Innovation Principle [12, 13], intended as a complementary, balancing principle to the Precautionary Principle, recognising the need to protect the society and the environment while also safeguarding a nation's ability to innovate. Both the underlying principles are the associated principles of proportionality and adaptation as applied to regulations and policies, designed to ensure that protecting the society from potential harm should, where possible, avoid stifling beneficial innovation. RI is expected to strike a balance across these principles.
2. It recognises that RI should be different at different stages of the innovation process and for different types of innovation (incremental innovation where the RI process can be a more ‘light touch’, and transformative innovation where stakeholder interests and values may be more contentious and the RI process more demanding) [1].
3. It takes on board the relative capacities of large and small companies to undertake RI and sets out clearly what can be expected from smaller companies with limited resources.
4. Recognising the need for a company to demonstrate publicly that it is behaving responsibly, it provides a framework to do so that is easily understood by companies of any size and their stakeholders. This builds on procedures that are already familiar to many companies, such as compliance with a social responsibility standard and using a risk assessment matrix as part of conventional project management.
5. It explicitly incorporates the need to be aware of and to comply with other existing regulations and standards.
6. Stakeholder engagement is an important part of RI, particularly for transformative innovations and any others that are potentially contentious, and guidance is also provided on how to conduct engagement initiatives.

PAS 440 involves an iterative process that revisits a company's RI monitoring at intervals appropriate to the speed of development of the innovation and takes account of any substantive changes in its properties or plans for future market targeting.

## 4 APPLICATION CASE STUDIES

This section describes two case studies carried out as dissertation projects by Masters students at Strathclyde University (A. Brown, Industrial Biotechnology Innovation Centre Collaborative Masters in Industrial Biotechnology) and the University of Edinburgh (I. Cabrera Lalinde, Masters in Management of the Bioeconomy, Innovation and Governance). Both involved spin‐out companies using synthetic biology‐related technologies to develop products for future markets. Both Masters projects were short (approximately 3 months) and so the extent of implementation of PAS 440 was limited. The case studies provided evidence to support the companies in further implementing PAS 440 and making it an integral part of their future development processes.

### 4.1 Case study 1—MiAlgae

In developing an understanding of MiAlgae's perspective on the PAS 440 framework, its impact on the company and its innovation plans, semi‐structured interviews were carried out with representatives of the organisation and of their key stakeholders, including the aquaculture industry, the whisky industry and not‐for‐profit organisations providing financial support.

#### 4.1.1 RI standard‐related challenges in this field

MiAlgae is a small industrial biotechnology company that is currently at a critical scale‐up stage, having recently received significant investment for the development of their product, an omega‐3‐rich algae‐based oil. The main market for MiAlgae's product is the aquaculture industry where it is required to meet the consumer standards for omega‐3 levels in farmed salmon and other carnivorous fish species. It is produced from co‐products of the whisky industry and would supplement the current supply of omega‐3 oils from wild‐caught fish, potentially having a significant impact on the environmental sustainability of the sector.

The public visibility of these consumer‐focused sectors requires careful consideration of any new product introduction or collaborative project [14]. This emphasises the importance of conducting innovation responsibly and the potential value of a widely recognised RI framework.

#### 4.1.2 Applying PAS 440—MiAlgae and stakeholder perspectives

MiAlgae saw itself as working to improve the sustainability of the aquaculture industry and noted that there is a risk, if they do not develop their product, of continued reliance on wild fish as a source of omega‐3 oils. They also saw themselves as making a contribution to a circular bio‐economy. The company has adopted the ISO 14001 Environmental Standard and ensures that its business partners are operating to ethical responsibility requirements, but they do not yet have any formalised goals in the RI area.

MiAlgae foresaw a potential reputational risk at the time of a future product launch, requiring a stronger focus on being able to demonstrate their sustainable practices to external stakeholders. Although PAS 440 is seen as having the potential to improve their reputation, brand value and overall stakeholder relationships, they could only justify the time spent on it if it had a definitive, measurable benefit for the organisation.

Some important customers in the aquaculture industry were more interested in product price than sustainability, but many of the companies wanting to collaborate with MiAlgae are also looking for sustainable solutions and have already addressed such issues. They have identified a future improvement by including sustainability criteria in their tendering process to ensure that their partners comply with the same general standards to which MiAlgae operates.

Exploring MiAlgae's current RI‐related initiatives and its relationships with stakeholders provided a baseline to build responsible innovation, based on PAS 440, into its own standard operating procedures and disseminating this along the supply chain in future.

Stakeholders commented on how MiAlgae's adoption of PAS 440 could have a positive impact on their own relationship with the company and with the wider industry. All agreed that adoption of the PAS 440 framework would deliver for MiAlgae all the benefits proposed above in Section 2, particularly based on its value as a tool to communicate with non‐technical stakeholders, such as supermarkets. It could usefully summarise much of the key information sought by these stakeholders.

Like MiAlgae, stakeholders also considered that the full value of the framework would only be realised once it had gained a wider recognition, given that it is only in the early stages of implementation. They thought that it would be particularly useful as a starting point for developing an approach to RI for smaller organisations with limited resources to fulfil its requirements. However, stakeholders also saw it as challenging to convince these organisations to commit the necessary resources when they cannot see it as directly relevant to the growth of their company.

Stakeholders involved in funding also noted that companies taking the time to plan their RI approach at an early stage in the product development would be able to avoid delays later on when applying for financial support by pre‐identifying potential benefits and hazards linked to their product introduction and if necessary suggesting mitigations.

#### 4.1.3 PAS 440 viability and opportunities for improvement

An important recommendation from the dissertation was that translational bodies such as the Industrial Biotechnology Innovation Centre (IBioIC) could champion the framework to assist small companies with limited resources to adopt PAS 440 as a standard. Such organisations already have strong relationships with innovative companies in their sector and could promote the framework more widely with a significant impact on awareness and its adoption.

### 4.2 Case study 2—Norfolk Plant Sciences

Norfolk Plant Sciences (NPS) is commercialising a purple tomato product, containing enhanced levels of anthocyanins, to be sold in the form of juice. It is a spin‐out company from the John Innes Centre (JIC) and The Sainsbury Laboratory (TSL), both independent research institutes specialising in plant and microbial science and genetics. Among many other claimed benefits, anthocyanins can reduce the incidence of cancer and improve cardiovascular function [15, 16] and regulatory approval for the product is being sought from the US Food and Drug Administration and Department of Agriculture. PAS 440 was viewed in this case study as a means to support JIC decision‐making on the creation of spinout companies for the commercialisation of its research, raising awareness of the social, ethical and regulatory challenges.

Interviewees included representatives of JIC and TSL who have been particularly involved in commercialising this technology, along with others from organisations used by JIC in supporting commercialisation of its research.

#### 4.2.1 RI standard‐related challenges in this field

Much of the research on plant science and agricultural biotechnology has focused on overcoming human chronic, non‐communicable diseases while also contributing to more sustainable and productive agricultural practices. Targeting phytonutrients such as anthocyanins can help to counteract the effects of poor or restricted diets in developed and developing countries.

Claims that the use of genetic technologies can improve both productivity and human health have been faced with citizen concerns about associated hazards, and the commercialisation of such products is potentially sensitive to public perception. Widespread misinformation about the products of genetic technologies have also influenced the expected marketability of new developments [17].

Adoption of plant genetic technologies to overcome societal and environmental challenges is accompanied by demands for more transparency, encouraging companies to make extra efforts to deal with uncertainties that might threaten their position in the market. Here PAS 440 provides a bridge to a better understanding of the balance between positive contributions and potential hazards from an innovation, significantly influencing how new products entering the market will be perceived.

#### 4.2.2 Applying PAS 440—NPS and JIC perspectives

Based on the PAS 440 RIF template, the aims of the project were to guide NPS and JIC to identify positive and negative elements related to this innovation, to provide insights on how to address any issues arising at different stages in the development process, and to document the outcomes of their RI approach. The work aimed to suggest areas where RI practices can contribute to advanced understanding of the relationships between companies and stakeholders relevant to positive or negative societal or environmental impacts.

Considering organisation‐level responsibility, JIC has an excellent history of engagement in RR on which to build RI, and interviewees recognised the importance of RI in moving to a more sustainable future for NPS. JIC has ensured that its commitment to diversity, equality and inclusion, and its vision to address global challenges through plant science, remain at centre stage as the product is commercialised. JIC interviewees agreed that many of the corporate‐level requirements for RI are already in place, but that some might not be simple to implement, for example, where the behaviour of potential partners in the future supply chain of a product is concerned. The proposed RI model could raise questions within JIC, NPS and its technology transfer company, on the economic and social value of the product, and there were also some concerns about JIC losing power over future development decisions made by NPS.

Considering product‐specific elements relevant to the purple tomato, given the involvement of innovative genetic technologies in the development of this product, it may attract negative attention from citizens and public interest activist groups. The case study used the PAS 440 RIF to categorise these elements, record the reasons for their inclusion and identify relevant stakeholders, and actions to be taken, to provide reassurance. The following positive outcomes of the innovation were identified: promoting the availability of affordable and nutritious food; educating local children; stimulating local businesses in developing countries; supporting sustainable production and consumption patterns; limiting cultivation to contained‐environment agriculture to remove the risks from genetic spread via pollination in open agriculture; and protecting communities against a range of non‐communicable diseases. Potential negative outcomes and issues included public and advocacy group concerns about innovative biotechnologies; the need for further assessment of the toxicology of the compounds present in the tomatoes; potential reputational damage from association with other companies in the supply chain and monitoring responsible behaviour of partners; and the need to assess the future environmental impact.

#### 4.2.3 PAS 440 viability and opportunities for improvement

Companies that proactively implement a framework to innovate responsibly could be better prepared for the challenges of a rapidly changing innovation ecosystem. An important benefit of implementing the RI standard was seen to be acquiring the ability to cope with and anticipate innovation‐related issues, including establishing protocols to move from a focus on RR to RI.

The flexibility and adaptability of PAS 440 will simplify the process of integrating different perspectives, given that it can be adapted to a changing environment, and can be used to align corporate and societal behaviours. Implementation of the RI standard prompted debates on changes that would have to be made within JIC to facilitate the creation of spinout companies and to cope with stakeholder demands in a competitive environment. In such cases, RI has a role to play in pitching to investors, for example, a seed fund to support the translation of JIC's innovation in agri‐food and health.

PAS 440 was also seen to have potential benefits to JIC and stakeholders by fostering wider recognition of the RI standard throughout the biotechnology industry sector.

PAS 440 could improve communication between stakeholders involved in the creation of spinout companies and guide the distribution of resources to facilitate commercialisation. Embedding responsible business practices within the corporate culture can translate into economic benefits resulting from reducing technical barriers to commercialisation of the innovation. It can also minimise costs arising from unforeseen circumstances related to stakeholder engagement and efficiency along the supply chain.

## 5 CONCLUSIONS AND NEXT STEPS

### 5.1 Conclusions relevant to the case studies

The two case studies presented here contribute to a body of knowledge on the usefulness of the PAS 440 Guide, what works and what should be changed or added to in future. Both case studies involved small companies, but MiAlgae had already received significant private investment, and was further along the commercialisation journey than NPS. NPS senior staff were still employed by their parent research institutes, giving them a different perspective on the future development process; also, the intended market for the purple tomato as a food for human consumption will create additional regulatory hurdles that will not apply to MiAlgae's omega‐3 oil and will take longer to negotiate, slowing down translation to a market.

Even though work had only recently begun, both companies could already see benefits in the adoption of PAS 440, and these were expected to become more significant as it became more widely adopted, creating greater future incentives for companies to adopt the RI standard. Despite these benefits, both MiAlgae and NPS foresaw some future hazards arising from association with other companies that might bring reputational damage or that did not appreciate the value of RI given their strong focus on getting a rapid return on their investment, the latter factor leading to concerns about the time and staff investment needed to deliver RI.

Given some of the companies' hesitancies, particularly those due to lack of resources, there was a greater willingness in both companies to implement the RI standard on a product‐by‐product basis, rather than to commit to adopting it across the board at this stage. The staged approach recommended by PAS 440 leaves space to allow a company to decide at what point to begin to consider RI. There might be some value in waiting until it is clear that a product has commercial potential before formally adopting an RI initiative. However, taking this on at an earlier stage would reduce or remove some of the uncertainty arising from investing in a product before evaluating RI‐related issues.

Here, as in most RI initiatives, stakeholder engagement was an important theme. It was seen as a two‐way process, allowing stakeholders to express their interests and expectations relevant to a product, and the company to have a role in framing stakeholder understanding of the product and its benefits and hazards. Particularly in the early stages of the product development, interaction with a broad range of stakeholders can enable a company to identify new markets for a product, or potential future problems that might arise in later stages. However, across all relevant stakeholders, there may not be a consensus on all the issues discussed. Each stakeholder will have a different perspective on the adoption and value of an innovation and the RI process can reassure stakeholders that all aspects of the innovation are being carefully considered.

Historically, stakeholder engagement has provided opportunities for some activist groups to exert a disproportionate influence on the public framing of innovative technologies and PAS 440 includes guidelines on engagement with stakeholders, taking on board the principle of equity and the onus on all parties involved in an engagement initiative to behave equally responsibly [7, p. 19].

### 5.2 The uptake and future development of PAS 440

In general, technology is neither good nor bad; it is how it is used that matters. Electricity lights and heats our homes, but an electric shock can kill. Medicines can cure ill health, but most are also poisonous. Pressure has been building over the past 10 years for the introduction of an RI standard that takes account of the joint needs of companies and their stakeholders and PAS 440 has an important role to play as an industry‐wide standard. It aims to support companies of all sizes to do the right thing by providing a framework to demonstrate the balance between potential benefits and harms and, if necessary, highlights actions to maximise the benefits and/or minimise the harms. Adoption of PAS 440 could improve communication between partners and stakeholders involved in the creation of spinout companies and guide the distribution of resources to facilitate commercialisation.

The PAS process enables a guide to be rapidly developed in order to fulfil an immediate need in industry, and PAS 440 has had an enthusiastic reception from companies across many innovative sectors of the economy. On the day of its launch in April 2020, it was downloaded from the BSI website more than a thousand times, a record for any BSI standard. At the time of writing in December 2020, it had reached 2700 downloads, more than the total for all the BSI standards supported by Innovate UK [18].

A series of webinars undertaken by BSI and the Knowledge Transfer Network in 2020, to support the cohort of the Innovate UK Fast Start COVID‐19 competition, introduced participants to PAS 440 and they considered it very digestible. Many participants had not engaged before with BSI and the RI Guide provided a practical support where the impact aligned well with the innovator's intentions. BSI informal discussions with major multinational companies have noted that they use similar processes to PAS 440 to assess responsibility and that the Guide would also be very useful to them. PAS 440 thus resonates with many companies already familiar with standards and is also an easy first step into understanding and using standards for those who are unfamiliar with them.

Two insights emerged from the case studies that should be considered for incorporation in future versions of the RI standard. First, it could be used to support decision‐making on whether or not to start a company, for example, providing information on the supply chain and stakeholder alignment and gaps in understanding but, as noted in Section 3, it should also avoid leading to a reputation for RI as ‘… the place where some innovation goes to die’. Second, the concept of integrating RI application along a supply chain, rather than applying it piecemeal to individual companies, could give that supply chain an advantage over competitors.

Taking up the points in Section 4 about the lack of resources for small companies to engage fully with PAS 440, accelerators could play a role as champions, providing early‐stage finance and mentoring. Similarly, IBioIC and other publicly funded translational bodies could provide a supportive environment for the adoption of the RI standard.

If many companies are actively using PAS 440, that could pave the way for further future developments as an accreditable British Standard, or a constituent part of the UK input into the development of a European or International Standard.

### 5.3 Links between innovation, regulatory reform and responsible innovation

As noted in the introduction, we are experiencing simultaneous expansion in the scale and speed of innovation across many sectors of the economy alongside recognition of the need for our regulatory systems to be more proportionate and adaptive to the properties of innovative technologies. As these trends continue, it will be increasingly essential for companies to demonstrate that they support, and are implementing a responsible approach to innovation and the availability of PAS 440 is already making a major contribution to this process. Other societal trends are moving in a similar direction to discussions on RI, for example, challenging traditional economic models and introducing a role for alternative value categories [19]. PAS 440 could be among the first of a series of standard tools to support better integration of values between industry and citizens.

## CONFLICT OF INTEREST

The authors based in BSI, Daniel Barlow and Matthew Chiles, have an interest in the future development of the PAS 440 standard.

## Supporting information

Supplementary Material
