# Supplementary material for: Responsible innovation: Its role in an era of technological and regulatory transformation
Source: Eng Biol. 2021 Mar 29;5(1):2–9. doi: 10.1049/enb2.12005 (PMC9996694; doi:10.1049/enb2.12005)
Supplement: Supplementary file 1 — Supplementary Material [file ENB2-5-2-s001.docx]

Responsible Innovation: its role in an era of technological and regulatory transformation.

Responsible Innovation

Authors’ names

Joyce Tait ^1*^, Alex Brown ^2^, Isabela Cabrera Lalinde ^1^, Daniel Barlow^3^, Matthew Chiles^3^, Paul Mason^4^.

Affiliation of authors:

^1^ Innogen Institute, University of Edinburgh, High School Yards, Edinburgh EH1 1LZ, UK

^2^ Strathclyde Institute of Pharmacy and Biomedical Sciences, University of Strathclyde, 161 Cathedral Street, Glasgow, G4 0RE, UK.

^3^ BSI, 389 Chiswick High Road, London, W4 4AL, UK

^4^ Innovate UK, Polaris House, North Star Avenue, Swindon, SN2 1FL, UK.

**Postal and e-mail address of the corresponding author:**

Innogen Institute, University of Edinburgh, High School Yards, Edinburgh EH1 1LZ, UK

joyce.tait@ed.ac.uk

**Funding:**

| **BEIS/BSI** | C13171, Oct. 2016 Edinburgh University ESRC Impact Grant, 2015/15 |
| --- | --- |

**Conflict of Interest:**

The authors based in BSI, Daniel Barlow and Matthew Chiles, have an interest in the future development of the PAS 440 standard.

**Permission to reproduce materials from other sources:**

None

**Data Availability Statement**

Research data are not shared.
